# Comparative transcriptome analysis reveals transcriptional regulation of anthocyanin biosynthesis in purple radish (*Raphanus sativus* L.)

**DOI:** 10.1186/s12864-024-10519-4

**Published:** 2024-06-20

**Authors:** Yi Liu, Chenchen Wang, Haidong Chen, Guoqiang Dai, Qiushi Cuimu, Wenjie Shen, Liwei Gao, Bo Zhu, Changbin Gao, Lunlin Chen, Daozong Chen, Xueli Zhang, Chen Tan

**Affiliations:** 1 College of Life Sciences, Ganzhou Key Laboratory of Greenhouse Vegetable, Gannan Normal University, Ganzhou, China; 2 Wuhan Vegetable Research Institute, Wuhan Academy of Agriculture Science and Technology, Wuhan, China; 3 Nanchang Branch of National Center of Oilcrops Improvement, Jiangxi Province Key Laboratory of Oil Crops Biology, Crops Research Institute of Jiangxi Academy of Agricultural Sciences, Nanchang, 330200 China

**Keywords:** Radish, Anthocyanin, Transcriptome, DFR, PAP1

## Abstract

Radish exhibits significant variation in color, particularly in sprouts, leaves, petals, fleshy roots, and other tissues, displaying a range of hues such as green, white, red, purple, and black. Although extensive research has been conducted on the color variation of radish, the underlying mechanism behind the variation in radish flower color remains unclear. To date, there is a lack of comprehensive research investigating the variation mechanism of radish sprouts, leaves, fleshy roots, and flower organs. This study aims to address this gap by utilizing transcriptome sequencing to acquire transcriptome data for white and purple radish flowers. Additionally, the published transcriptome data of sprouts, leaves, and fleshy roots were incorporated to conduct a systematic analysis of the regulatory mechanisms underlying anthocyanin biosynthesis in these four radish tissues. The comparative transcriptome analysis revealed differential expression of the anthocyanin biosynthetic pathway genes *DFR*, *UGT78D2*, *TT12* and *CPC* in the four radish tissues. Additionally, the WGCNA results identified *RsDFR.9c* and *RsUGT78D2.2c* as hub genes responsible for regulating anthocyanin biosynthesis. By integrating the findings from the comparative transcriptome analysis, WGCNA, and anthocyanin biosynthetic pathway-related gene expression patterns, it is hypothesized that genes *RsDFR.9c* and *RsUGT78D2.2c* may serve as pivotal regulators of anthocyanins in the four radish tissues. Furthermore, the tissue-specific expression of the four copies of *RsPAP1* is deemed crucial in governing anthocyanin synthesis and accumulation. Our results provide new insights into the molecular mechanism of anthocyanin biosynthesis and accumulation in different tissues of radish.

**Supplementary Information:**

The online version contains supplementary material available at 10.1186/s12864-024-10519-4.

## Introduction

Anthocyanins, which are flavonoid secondary metabolites, play a pivotal role as natural pigments in plants. They impart red, purple, or blue colors to different plant tissues, including leaves, stems, roots, flowers, and fruits, thereby contributing to plant growth and development [19, 29]. Moreover, these compounds serve as attractants for pollinators and aid in seed dispersal [10]. Furthermore, anthocyanins have been extensively studied for their involvement in plant responses to both biotic and abiotic stresses, encompassing UV protection, pigmentation, and defense mechanisms [30, 38]. The antioxidant properties and associated health benefits of anthocyanins have attracted considerable attention. These compounds, when included in the diet, not only act as safe and non-toxic natural food colorants but also exhibit potent free radical scavenging activity [1, 27]. This activity has the potential to reduce the risk of certain cancers, cardiovascular diseases, and other chronic ailments [16, 38]. Consequently, the cultivation of vibrantly colored vegetable varieties enriched with anthocyanins holds substantial commercial value. This endeavor aligns closely with the goals of breeders, as it addresses both consumer preferences and the pursuit of healthier dietary choices.

Since its discovery in 1986, the *C1* gene has been identified as a key regulator in the biosynthesis of anthocyanin pigments in the aleurone layer of maize endosperm [6]. Subsequently, numerous studies have elucidated the biosynthetic and transcriptional regulation mechanisms of anthocyanins in various plant species [13, 15, 21, 26, 29, 41, 42]. Genes related to the anthocyanin biosynthetic pathway mainly include early biosynthetic genes (EBGs) *phenylalanine ammonia-lyase* (*PAL*), *cinnamate-4-hydroxylase* (*C4H*), *4-coumarate CoA ligase 4* (*4CL*), *chalcone synthase* (*CHS*), *chalcone isomerase* (*CHI*), *flavanone 3’-hydroxylase* (*F3’H*), and late biosynthetic genes (LBGs) *dihydroflavonol reductase* (*DFR*), *anthocyanin synthetase*/ *leucoanthocyanin dioxygenase* (*ANS* /*LDOX*), and *UDP-flavonoid glucosyltransferase* (*UFGT*). Concurrently, extensive research has demonstrated that the expression of anthocyanin biosynthetic genes is controlled at the transcriptional level, involving transcription factors from the MYB, bHLH, and WD40 protein families [9, 29, 32]. In the phenylpropanoid biosynthetic pathway, the regulation of *PAL*, *C4H*, and *4CL* expression is mediated by MYB transcription factors *MYB3*, *MYB4*, *MYB7*, and *MYB32* [7, 12, 22]. Similarly, in the flavonoid biosynthetic pathway, the expression of *CHS*, *CHI*, and *F3'H* is controlled by MYB transcription factors *MYB11*, *MYB12*, and *MYB111* [24, 28, 31]. Lastly, in the anthocyanin biosynthetic pathway, the regulation of *DFR*, *ANS* (*LDOX*), and *UFGT* expression is orchestrated by the MBW transcriptional regulatory complex, which is composed of MYB transcription factors *MYB75*, *MYB90*, *MYB113*, *MYB114*, and bHLH transcription factors *TT8*, *GL3*, *EGL3*, and WAD40 proteins [9, 14, 32, 36].

Radish (*Raphanus sativus* L., RR, 2n = 18) is a highly nutritious vegetable, abundant in phenols, vitamin C, chlorophyll, carotenoids, and glucosinolates, which confer potential health benefits to individuals. Consequently, it is extensively cultivated and consumed globally [43]. The roots, leaves, and flowers of radish exhibit considerable diversity in terms of color, shape, and size. Notably, the wide range of color variations has emerged as a prominent characteristic that appeals to consumers. Previous studies have investigated the color variation in various tissues of radish, including sprouts, leaves, and roots. Muleke et al. [17] utilized four inbred lines of radish to conduct a comparative transcriptome analysis at three different developmental stages (10DAS, 30DAS, 50DAS), there findings indicate that the primary regulatory factor in radish anthocyanin biosynthesis is *RsUFGT*. Furthermore, they observed a close association between the transcript levels of *RsUFGT*, *RsF3H*, *RsANS*, *RsCHS3*, and *RsF3'H1* and the overall concentration of anthocyanins [17]. Upon subjecting radish sprouts to UV-A treatment, there was a significant increase in anthocyanin content, through comparative transcriptome sequencing analysis, the transcript levels of transcriptional regulators related to the MYB-bHLH-WD40 complex were consistent with the observed concentration of anthocyanins [40]. In the study conducted by Pu et al. [23], it was observed that the expression levels of the structural genes *PAL4*, *4CL4*, *CHS*, *F3H*, *DFR*, *ANS*, *OMT1*, as well as the transcription factors *TT8*, *CPC*, and *MYB114*, were significantly elevated in purple leaves. Similarly, Gao et al. [8] found that in radish fleshy roots, the transcription factors *RsMYB_9*, *RsERF070*, and the anthocyanin synthesis-related genes *RsCHS*, *RsCHI*, *RsANS*, *RsMT2-4*, *RsUF3GT*, *RsUFGT78D2-like*, and *RsUDGT-75C1-like* play crucial roles in anthocyanin biosynthesis in radish varieties. In the context of root-skin color variants, the *CHS* gene exhibits specific expression in red skin, suggesting its potential role as a crucial regulator in the accumulation of red pigment in red-skinned radish. Furthermore, the expression levels of transcription factors *MYB1/2/75*, bHLH (*TT8*), WD40, and the structural genes involved in the anthocyanin biosynthetic pathway are significantly higher in red skin compared to white skin [35]. In a recent study by Zhang et al. [37, 39], a comparative transcriptome analysis was conducted using six radish materials with varying skin and flesh colors, aiming to identify key genes responsible for anthocyanin biosynthesis. The findings of this study demonstrated that *RsTT4*, *RsC4H*, *RsTT7*, *RsCCOAMT*, *RsDFR*, and *RsLDOX* were significantly upregulated in the red- and purple-colored accessions. The metabolic and transcriptome analysis findings of the dark red taproot of radish indicate that *RsCHS* and *RsDFR* exhibit the highest up-regulation in this particular taproot. Additionally, the heightened co-expression of *RsMYB1* and *RsMYB2* potentially plays a role in the development of the dark red coloration [11]. However, the molecular underpinnings of radish flower color variation remain unexplored, and there exists a dearth of comprehensive investigations into the mechanisms governing color variation in radish hypocotyl, leaves, fleshy roots, and petals. In this study, the most recent published radish reference genome information (Xinlimei) was utilized as a reference. Sequencing and transcriptome data were gathered from radish hypocotyl, leaves, fleshy roots, and petals to perform a comparative analysis. The findings revealed that 10 genes exhibited significant differential expression across all tissues. The results of GO enrichment and KEGG annotation indicated that these differentially expressed genes were enriched in the pathways associated with flavonoid metabolism and anthocyanin metabolism. Specifically, among the 10 genes that were differentially expressed, four exhibited a close association with anthocyanin biosynthesis. Following this, we employed the known anthocyanin-related genes of Arabidopsis thaliana to perform a comprehensive search for genes associated with the anthocyanin biosynthetic pathway in radish at a genome-wide level. A total of 103 genes were successfully identified, with several genes displaying tandem duplications. Additionally, we conducted weighted correlation network analysis (WGCNA) utilizing transcriptome data from four distinct tissues, encompassing 24 sets of gene expression data related to anthocyanin synthesis. The findings indicate that *RsDFR.9c* and *RsUGT78D2.2c* may serve as hub genes in the biosynthesis of anthocyanins. Moreover, by combining comparative transcriptome data and WGCNA results, we have constructed potential regulatory patterns for anthocyanin production in the four tissues of radish. The distinct synthesis and accumulation of anthocyanins in various radish tissues may be intricately linked to the tissue-specific expression of distinct copies of *RsPAP1*. Consequently, our investigation contributes to the advancement of knowledge regarding the genetic and molecular mechanisms that govern the tissue-specific biosynthesis and accumulation of anthocyanins in radish.

## Materials and methods

### Plant materials

The purple-flowered and white-flowered radish inbred lines used in this study were grown at the Wuhan Academy of Agriculture Science and Technology experimental base. Under the same cultivation conditions, white-flowered and purple-flowered radish were planted in the experimental plot, and flower buds (about 3 mm in length) to be opened were selected for sampling during the flowering period. Three biological replicates were taken from each material. All samples were then frozen in liquid nitrogen and stored at 80 °C for RNA extraction. The RNA-seq data of hypocotyl and fleshy roots were collected from National Center for Biotechnology Information (NCBI, https://www.ncbi.nlm.nih.gov/) with the following biological projects PRJNA388018 (hypocotyl), PRJNA810281 (fleshy roots), PRJNA810914 (fleshy roots). The RNA-seq data of leaves were collected from National Genomics Data Center (https://ngdc.cncb.ac.cn/gsa/browse/CRA008485) under accession ID CRA008485.

### Transcriptome analysis of the purple and green/white radish tissues

The raw data obtained from the NCBI was initially converted into fastq format using the SRA toolkit. Then, all raw data processed with default parameters using trimmatic (v0.39, [2]). Subsequently, the RNA-seq clean reads were aligned to the reference genome of radish (Xinlimei, http://brassicadb.cn/#/) using the HISAT2 software (v2.1.0, [20]). The obtained millions of fragments per thousand bases (FPKM) values were then calculated using StringTie (v2.1.1, [20]). The expression histogram was generated using Excel, while the heat map was generated using TBtools software (v1.133, [4]). In addition, Gene Ontology (GO) and KEGG pathway functional enrichment analyses of DEGs were significantly enriched in GO terms and metabolic pathways at Bonferroni-corrected *P*-value ≤ 0.05 compared with the whole-transcriptome background. GO functional enrichment and KEGG pathway analysis were carried out by Goatools (https://github.com/tanghaibao/Goatools) and KOBAS (http://kobas.cbi.pku.edu.cn/home.do). The 25 enrichment items with the highest correlation (Q-value) were used to draw the GO enrichment map, and the map was generated using TBtools software (v1.133, [4]).

### Genome-wide identification of genes related to anthocyanin biosynthesis in radish

Here, all the protein sequences and CDS sequences radish (Xinlimei) were obtained from the Brassicaceae Database http://www.brassicadb.cn/#/ [37, 39]. The genes related to anthocyanin biosynthesis from Arabidopsis was selected as the reference for identifying homologous genes in the radish. To ensure accurate identification homologous genes of anthocyanin biosynthesis related genes in radish, the following steps were primarily employed. Firstly, initial searches were conducted using local BLASTP and local BLASTN algorithms, with a significance threshold of E < 1e—20. Secondly, the screening process involved identifying candidate genes that exhibited a consistency rate exceeding 65% and a coverage rate surpassing 60%.

### Construction of co-expression network modules using association analysis modules and phenotypes

To explore the metabolite fluxes of the anthocyanin biosynthetic pathway and identification of hub genes regulating anthocyanin biosynthesis in radish, we employed the weighted gene co-expression network analysis (WGCNA) to establish a gene co-expression network for the transcriptional regulation of anthocyanin in four tissues of radish. The construction of co-expression networks was carried out using the WGCNA software package, specifically version R-4.3.1. Subsequently, correlation analysis was performed to determine the correlation between each co-expression module and the collected tissue data of radish, enabling the identification of hub genes associated with anthocyanin. All 24 sets of transcriptome expression data of anthocyanin biosynthesis-related genes in four radish tissues were then utilized to construct a co-expression network module. For the detailed method, refer to the articles of Chen et al. [4]. Finally, the gene regulatory network data analyzed by WGCNA was imported into Cytoscape (v 3.80, [25]) for visual display.

### RNA extraction, and qRT-PCR analysis

The green and purple hypocotyls, leaves, roots, and the white and purple flowers with three biological replicates were collected, all samples were collected and immediately frozen in liquid nitrogen for RNA extraction and qRT-PCR analysis. eight genes (*RsDFR.9c*, *RsUGT78D2.2c*, *RsCPC.1c*, *RsTT8.9c*, *RsPAP1.5c*, *RsPAP1.7c1*, and *RsPAP1.7c2*) have been used for qRT-PCR analysis, and the Actin3 gene as an internal reference gene control. The primers information was list in Supplementary Table S6. For the detailed methods, please refer to Chen et al. [4]. All materials of this research were collected from Gannan Normal University research base.

## Results

### Identification of differentially expressed genes (DGEs) in different tissues of radish

**Fig. 1:**
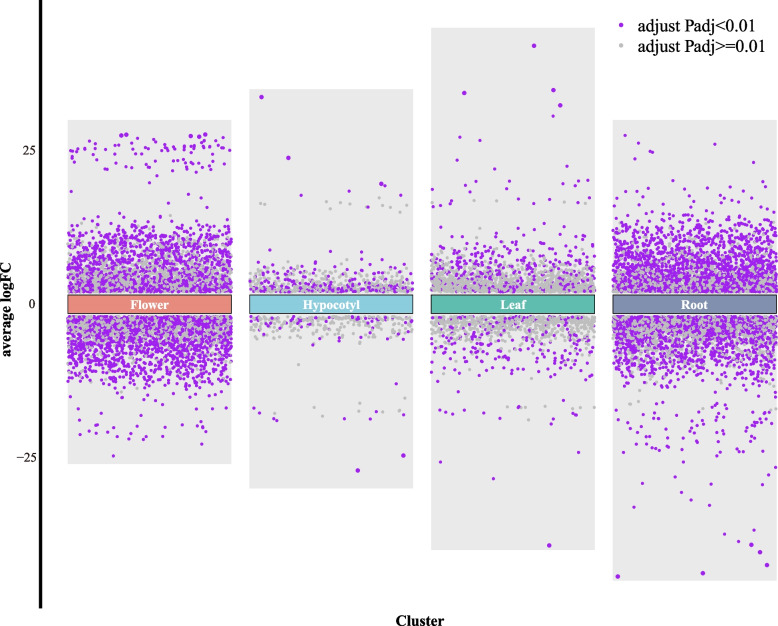
Volcano plot of differentially expressed genes in four radish tissues

The radish plant exhibits a diverse range of organ and color variations, rendering it a valuable specimen for investigating the mechanisms underlying color formation and regulation. In order to elucidate the genome-wide gene expression patterns associated with purple pigmentation in different radish tissues, we performed a comparative transcriptome analysis encompassing hypocotyls, leaves, radish taproots, and petals. The application of principal component analysis (PCA) yielded distinct separation among these various radish tissues (Supplementary Fig. 1). Significantly, the three biological replicates of the same tissue, albeit with distinct colors, were successfully clustered together, thereby emphasizing the reproducibility and consistency of the RNA-seq data. Additionally, we conducted a differential expression analysis using DESeq2, whereby genes were deemed significantly differentially expressed if they displayed |log2FC|≥ 2 and a *P*-value ≤ 0.01. Our expression analysis unveiled that, out of the 44109 annotated genes in radish (reference genome RS00, Xin-li-mei), 25152, 27607, 28209, and 27607 genes exhibited differential expression in the hypocotyl, leaf, radish taproot and flower, respectively (Table 1, Fig. 1).

**Table 1:** Number of differentially expressed genes in different tissues

Tissue	Significant			Not-significant	Total
	Up-regulated	Down-regulated	Total		
Hypocotyl	248	78	326	24826	25152
Leaf	453	267	720	26887	27607
Root	2110	1756	3866	24343	28209
Flower	1534	1863	3397	24210	27607

Specifically, the number of significantly differentially expressed genes (DEGs) in purple tissues compared to green/white tissues varied across different plant organs. In the hypocotyl, leaf, radish taproot, and flower, there were 326, 720, 3866, and 3397 DEGs, respectively (Table 1, Fig. 1, Supplementary Table S1). Further analysis revealed that there were 114 genes specifically expressed in the hypocotyl, 331 genes specifically expressed in the leaf, 2636 genes specifically expressed in the radish taproot, and 2213 genes specifically expressed in the flower (Supplementary Fig. 2). Notably, there were 10 genes that exhibited differential expression in all four plant organs, namely the hypocotyl, leaf, radish taproot, and flower (Supplementary Fig. 2, Supplementary Table S2).

### GO and KEGG enrichment analysis of DGEs

**Fig. 2:**
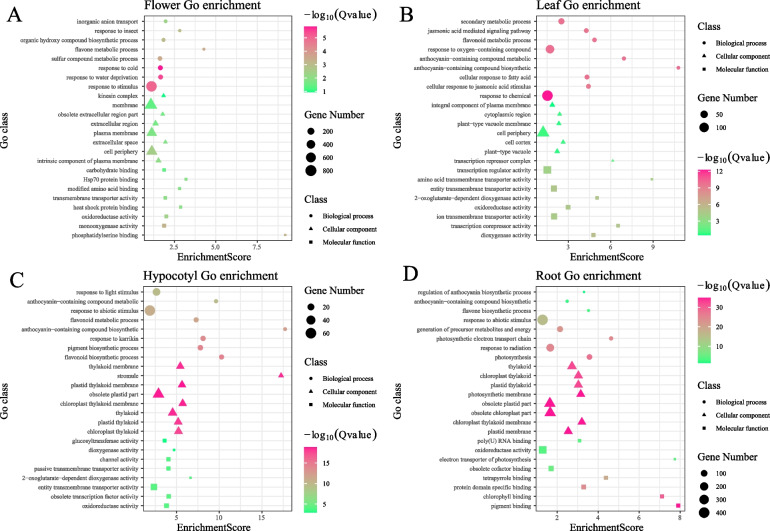
Gene Ontology (GO) enrichment analysis of the DEGs between four tissues of radish. **A** GO enrichment analysis of flower DEGs; **B** GO enrichment analysis of llower DEGs; **C** GO enrichment analysis Hypocotyl DEGs; **D** GO enrichment analysis of root DEGs

To further assess the biological functionalities of differentially expressed genes (DEGs) in four distinct tissues of radish, we conducted GO and KEGG enrichment analyses on the DEGs identified in these tissues and obtained 25 enrichment items with the highest Q-value (Fig. 2, Supplementary Fig. 3). Notably, in petals, DEGs exhibited the most significant enrichment score in pathways related to phosphatidylserine binding and flavone metabolic processes (Fig. 2A). Conversely, in leaves, DEGs displayed the highest enrichment score in pathways associated with anthocyanin-containing compound biosynthesis, amino acid transmembrane transporter activity, and anthocyanin-containing compound metabolism (Fig. 2B). In the hypocotyl, differentially expressed genes (DEGs) are primarily involved in the biosynthesis of anthocyanin-containing compounds, with the stromule pathway exhibiting the highest enrichment score (Fig. 2C). In the root, DEGs are predominantly enriched in pathways related to pigment binding, biosynthesis of anthocyanin-containing compounds, and flavone biosynthesis (Fig. 2D). KEGG annotation analysis reveals that DEGs in the hypocotyl, leaf, radish taproot, and flower are enriched in metabolic pathways associated with flavonoid biosynthesis, phenylpropanoid biosynthesis, and isoflavonoid biosynthesis (Supplementary Fig. 3).The results of the GO functional enrichment and KEGG pathway annotation analysis indicate that the different color tissues of radish hypocotyl, leaf, radish taproot, and flower exhibit significant enrichment in the flavonoid/anthocyanin metabolism pathway. This finding suggests that the biosynthesis and accumulation of flavonoids/anthocyanins may play a crucial role in the observed color variation in these tissues. Furthermore, the GO functional enrichment and KEGG pathway annotation analysis of the 10 differentially expressed genes (DEGs) shared by hypocotyl, leaf, radish taproot, and flower tissues revealed significant enrichment of *Rs0* × *7c033250*, *Rs0* × *9c039896*, *Rs0* × *2c007983* and *Rs0* × *1c000114* in the flavonoid/anthocyanin biosynthesis pathway (Supplementary Table S2).

### Identification of anthocyanin biosynthesis-related genes in radish

**Fig. 3:**
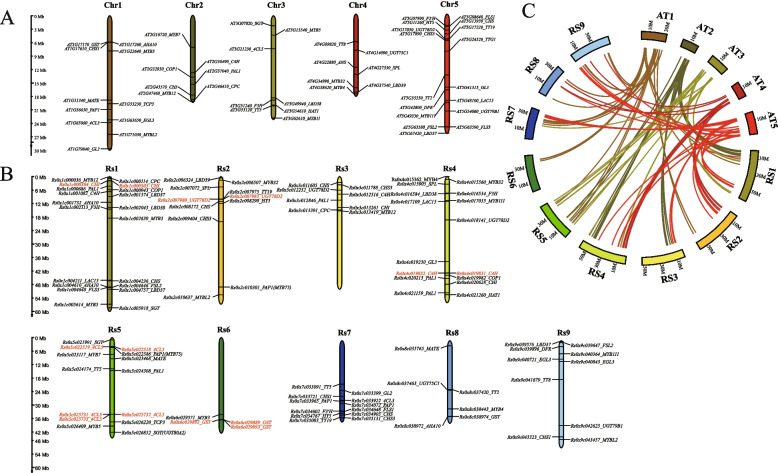
Distribution of anthocyanin biosynthesis-related genes in Arabidopsis and radish and their collinear relationship. **A** Distribution of anthocyanin biosynthesis-related genes on *Arabidopsis* chromosomes; **B** Distribution of anthocyanin biosynthesis-related genes on radish chromosomes; **C** Collinearity of anthocyanin-related genes in *Arabidopsis* and radish. Bars represent chromosomes, and the relative positions of anthocyanin biosynthesis-related genes are marked on the chromosomes, and the scale on the left shows the physical distance of the chromosomes

Both Radish and *Arabidopsis thaliana* belong to the Brassicaceae family, sharing a common ancestor and exhibiting a close relationship [18]. The structural genes and transcriptional regulators associated with the anthocyanin biosynthetic pathway in Arabidopsis have been extensively studied and characterized [9, 33]. By employing a method that combines homologous gene identification with collinearity analysis, we successfully identified a total of 103 genes related to anthocyanin biosynthesis in radish (Fig. 3, Supplementary Table S3). Among the identified genes, 48 were determined to possess homology with the 50 known anthocyanin biosynthesis-related genes in *Arabidopsis* (Fig. 3A, Supplementary Table S3). Notably, *PAL1* and *4CL5* exhibited the most significant degree of homology, with each gene having up to five homologous counterparts. In contrast, no homologous genes for *TTG1* and *MYB11* were detected. The total count of genes associated with anthocyanin biosynthesis in radish amounts to 103, distributed across nine chromosomes. The number of these genes varies on each chromosome, with 20 on Rs1, 12 on Rs2, eight on Rs3, 17 on Rs4, 14 on Rs5, four on Rs6, 12 on Rs7, six on Rs8, and ten on Rs9 (Fig. 3B, Supplementary Table S3). Significantly, all five homologous genes of *4CL5* are situated on chromosome Rs5. Specifically, *Rs0* × *5c022518* and *Rs0* × *5c022519* are arranged in tandem on one arm of chromosome Rs5, while *Rs0* × *5c025731*, *Rs0* × *5c025732*, and *Rs0* × *5c025733* are arranged in tandem on the opposite arm of chromosome Rs5 (Fig. 3B, C, Supplementary Table S4). A comparable pattern is observed for *C4H* (*Rs0* × *4c019831*, *Rs0* × *4c019832*), *CHI* (*Rs0* × *1c000304*, *Rs0* × *1c000305*), and *UGT78D2* (*Rs0* × *2c007980*, *Rs0* × *2c007983*) genes, as well as *GST* (*Rs0* × *6c029889*, *Rs0* × *6c029892*, *Rs0* × *6c029893*) genes (Fig. 3B, C, Supplementary Table S4). It is noteworthy to mention that Brassica species have experienced whole-genome tripling events throughout their evolutionary trajectory in comparison to Arabidopsis [5]. The observed tandem duplications in radish may potentially arise as a consequence of the aforementioned whole-genome tripling event.

### Analysis of anthocyanin related gene expression patterns in different tissues and colors of radish

**Fig. 4:**
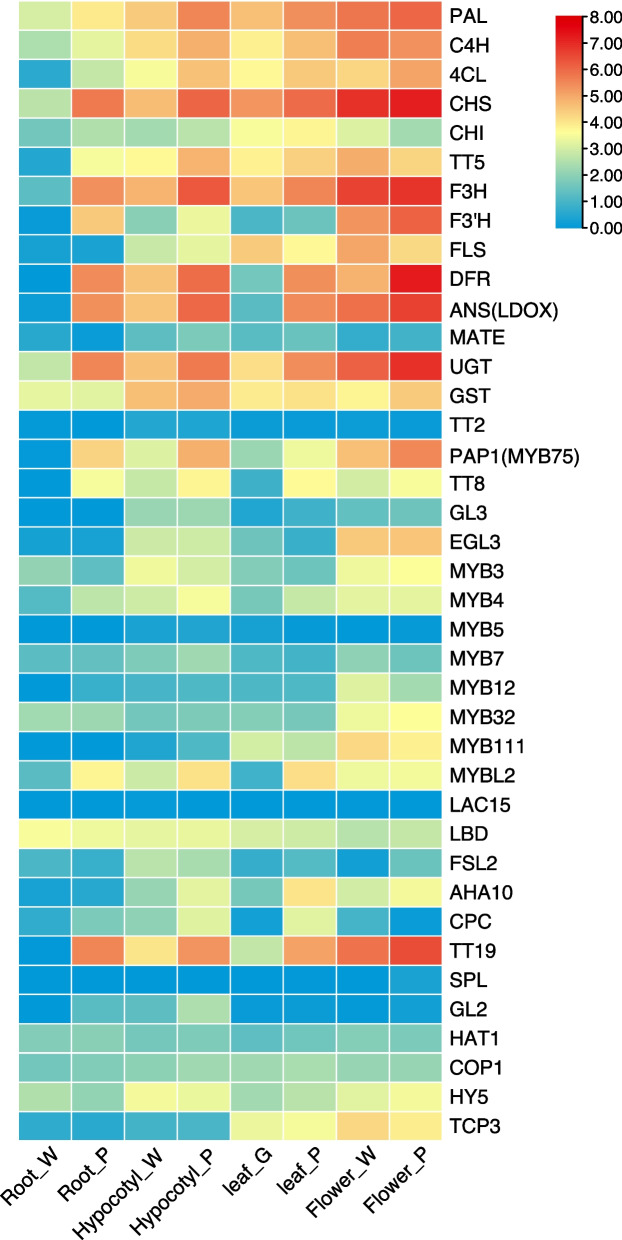
The expression patterns of anthocyanin-related genes in four tissues of radish were analyzed using a heat map that represents the FPKM values of these genes. The colors on the heat map, ranging from red to pink and white, indicate the expression levels from high to low

To further investigate the expression patterns of genes associated with the anthocyanin biosynthesis pathway in various tissues of radish, including hypocotyl, leaf, taproot, and flower, we utilized comparative transcriptome data from these tissues. This data was used to construct a heat map illustrating the expression levels of genes involved in the anthocyanin metabolism pathway (Fig. 4). Notably, early anthocyanin biosynthetic genes (EBGs) such as *PAL*, *C4H*, *4CL*, *CHS*, *CHI*, and *F3'H* exhibit a certain level of expression in both white/green and purple tissues. This can be attributed to the fact that anthocyanins are also involved in flavonol biosynthesis (Fig. 4, Supplementary Table S5). In this pathway, the elevated expression of *flavonol synthase* (*FLS*), a crucial catalytic enzyme for flavonol synthesis, in white flowers and green leaves provides strong evidence for this assertion (Fig. 4). Conversely, the late anthocyanin biosynthetic genes (LBGs) *DFR*, *ANS* (*LDOX*), and *UGT* exhibited significantly higher expression levels in purple tissues (Fig. 4). Among the transcriptional regulators, the expression pattern of MYB3, MYB4, MYB7, MYB12, MYB32, and MYB111, which are genes regulated by EBGs, closely resembles that of EBGs and is observed to a certain degree in both white/green and purple tissues. The regulatory genes *PAP1* and *TT8* of LBGs exhibited a significantly higher expression in purple tissues, aligning with the expression pattern of LBGs. Furthermore, we observed a significant up-regulation of glutathione transferase *TT19* in purple tissues, which displayed a similar expression trend to that of LBGs (Fig. 4).

### Co‑expression network analysis and identification of key regulatory genes

**Fig. 5:**
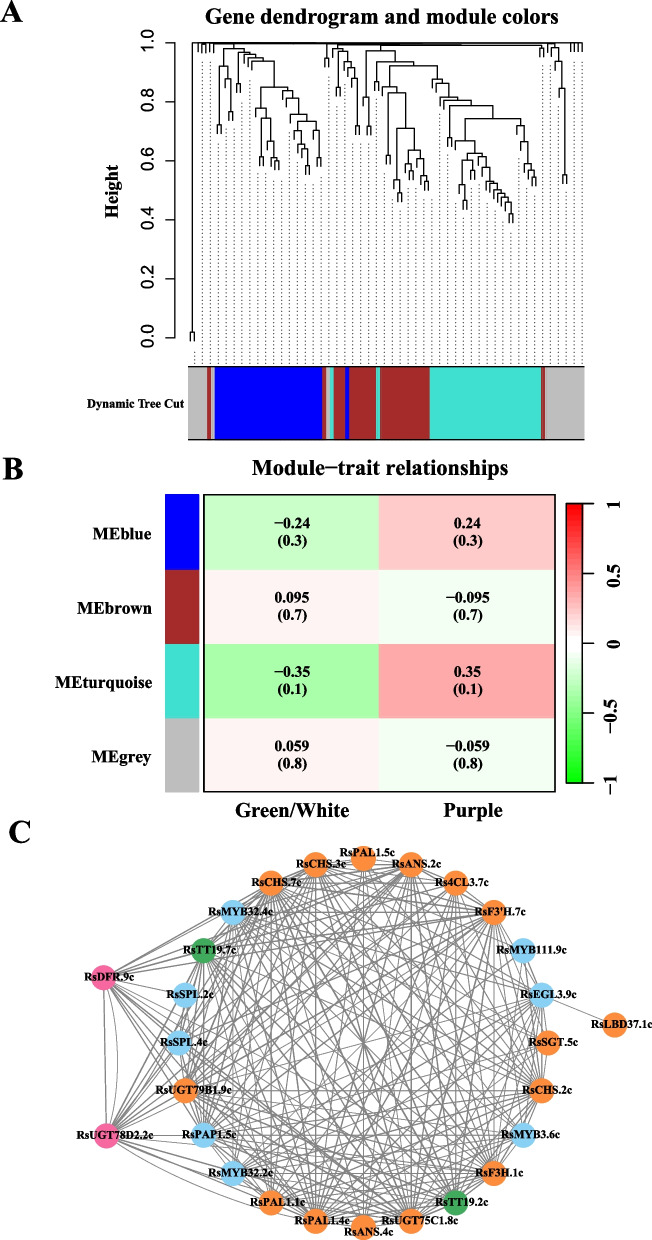
Co-expression analysis of genes related to anthocyanin biosynthesis was conducted in four tissues of radish. **A** Modular hierarchical clustering: The co-expression modules are depicted in different colors, while gray modules indicate no correlation between genes. **B** Module-to-sample correlation heatmap: Correlation analysis was performed between the co-expression modules of various genes associated with anthocyanin biosynthesis in different tissues. The numbers above the heat map indicate the Pearson correlation coefficient (r) values. **C** Cytoscape representation of co-expression network of the hub gene in the ‘MEturquoise’ module

Based on the expression trend of genes associated with the anthocyanin biosynthetic pathway, all genes were categorized into four modules using the Weighted Gene Co-expression Network Analysis (WGCNA) approach, one of which was an unclustered gray module (Fig. 5A). Notably, the 'MEturquoise' module exhibited the strongest positive correlation with the purple phenotype (*r* = 0.35, *p* = 0.1), as well as the strongest negative correlation with the green/white phenotype (*r* = -0.35, *p* = 0.1), suggesting a potential close association between the genes within this module and anthocyanin biosynthesis (Fig. 5B). The ‘MEturquoise’ module exhibits the most significant positive correlation in the purple phenotype (*r* = 0.35, *p* = 0.1) and the most significant negative correlation in the green/white phenotype (*r* = -0.35, *p* = 0.1). This suggests that the genes within this module may have a close association with anthocyanin biosynthesis (Fig. 5B). To further investigate the connection between the genes within the module and the screening hub genes (genes with high connectivity), a correlation network was constructed for the ‘MEturquoise’ module. In the 'MEturquoise' module, a total of 27 genes associated with the synthesis of anthocyanin biopathways exhibited a significant level of expression correlation. These genes include *PAL1*, *4CL3*, *CHS*, *F3H*, *DFR*, *ANS*, *UGT*, *SGT* and *LBD37* (structural genes), *SPL*, *MYB3*, *MYB32*, *MYB111*, *PAP1* and *EGL3* (transcription factors), and TT19 (transporter). Notably, the comparative transcriptome analysis of four tissues revealed differential expression of *RsUGT78D2.2c* and *RsDFR.9c* between purple and green/white samples. This finding suggests that these two genes may play a crucial role in regulating anthocyanin biosynthesis and accumulation in various tissues of radish.

### Transcriptional regulation pattern of anthocyanin biosynthesis in four radish tissues

**Fig. 6:**
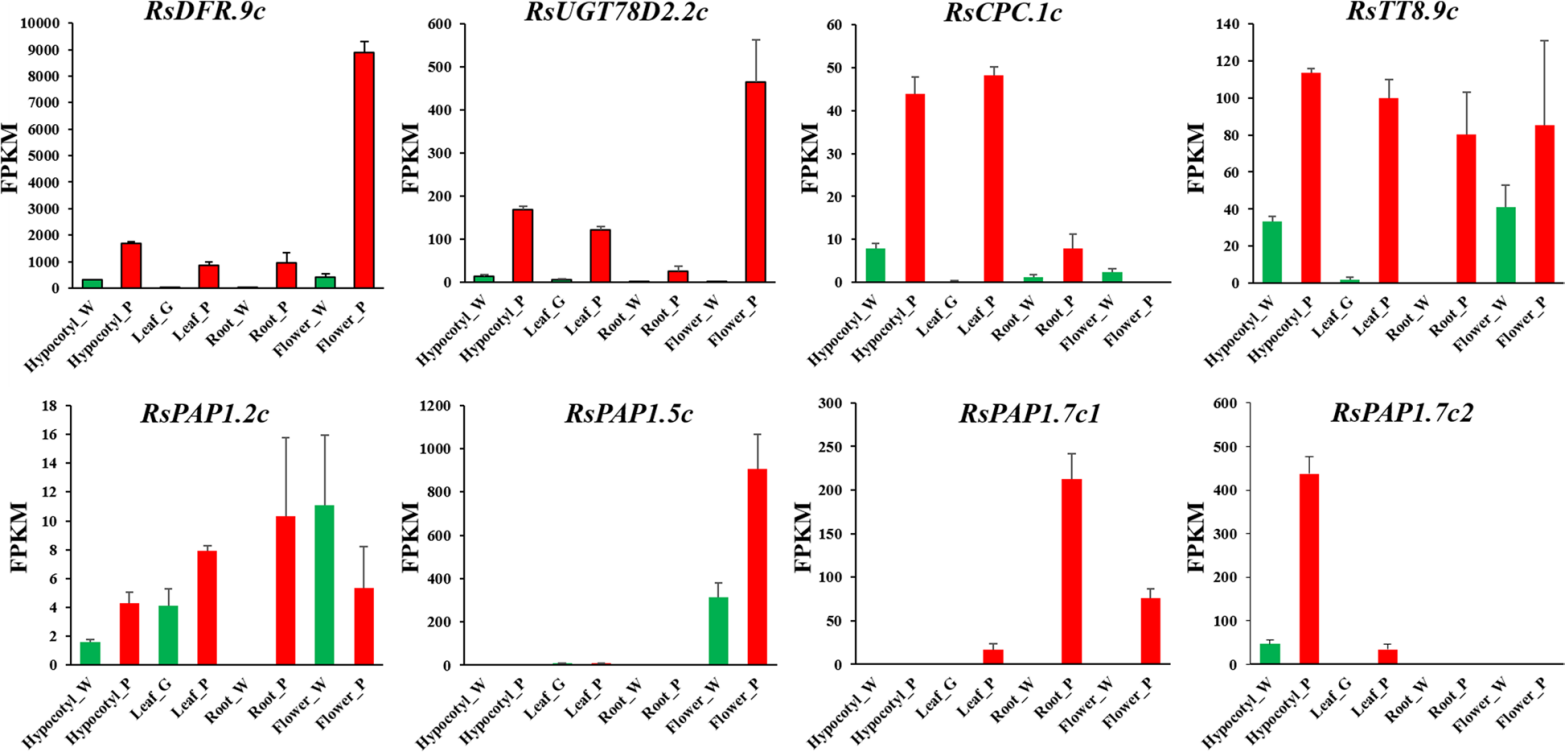
Expression analysis of eight hub genes in radish four different tissues. The FPKM (Fragments Per Kilobase Million) value obtained from transcriptome sequencing data analysis was used to represent the expression pattern of the hub genes in different tissues, both four different tissues with three biological replicates

To investigate the expression pattern of hub genes identified through comparative transcriptome and WGCNA analysis, we conducted expression calculations for *RsDFR.9c*, *RsUGT78D2.2c*, *RsCPC.1c*, *RsTT8.9c*, *RsPAP1.2c*, *RsPAP1.5c*, *RsPAP1.7c1*, and *RsPAP1.7c2* using transcriptome sequencing data from four different radish tissues (Fig. 6). The findings revealed a significant up-regulation of *RsDFR.9c*, *RsUGT78D2.2c*, *RsCPC.1c*, *RsTT8.9c*, *RsPAP1.5c*, *RsPAP1.7c1*, and *RsPAP1.7c2* in purple tissues, whereas *RsPAP1.2c* exhibited expression primarily in white petals of flowers, with a higher expression level compared to purple tissues. Among the four homologous copies of *RsPAP1*, it is noteworthy that the expression levels of *RsPAP1.5c*, *RsPAP1.7c1*, and *RsPAP1.7c2* exhibit distinct tissue expression specificity, in addition to *RsPAP1.2c*. Specifically, *RsPAP1.5c* demonstrates high expression in floral organs, while *RsPAP1.7c1* displays high expression in fleshy roots and also exhibits differential expression in flowers and leaves. Furthermore, *RsPAP1.7c2* exhibits high expression in the hypocotyl (Fig. 6).

**Fig. 7:**
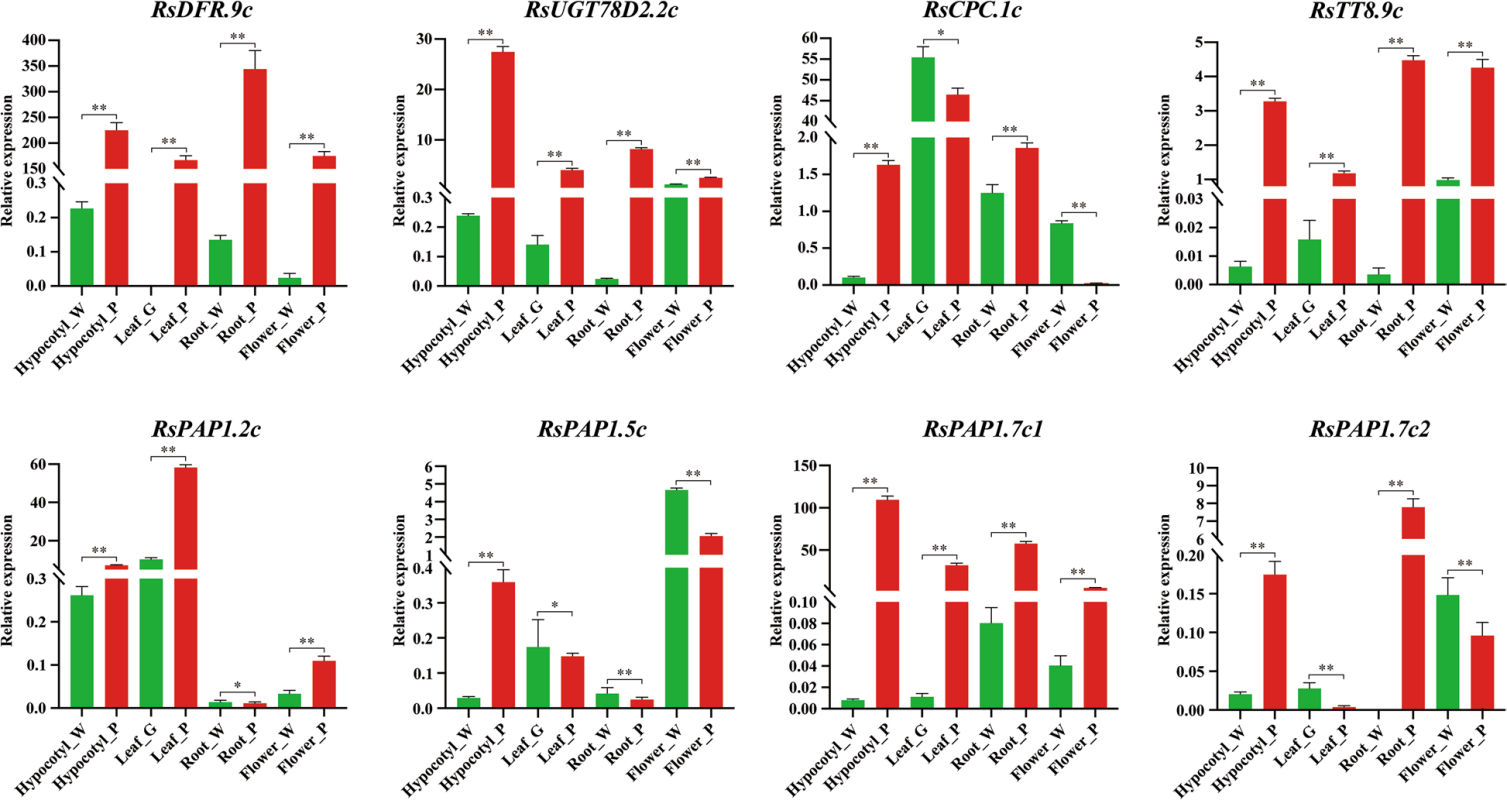
qRT-PCR validation of eight hub genes related to anthocyanin synthesis regulation in hypocotyl, leaves, fleshy roots, and petals. For qRT-PCR analysis, both white/green or purple sprouts, leaves, fleshy roots and flowers samples are with three biological replicates, the point represents the mean value of three technical replicates in a representative biological experiment, the error bars indicate s.d, student’s t-test, **P < 0.01, *P < 0.05

To further elucidate the transcriptional regulation pattern of anthocyanin biosynthesis in the four tissues of radish, we employed comparative transcriptome data and WGCNA analysis to construct a regulatory pattern map of key genes involved in anthocyanin biosynthesis in these tissues (Fig. 7). In both radish sprouts and leaves, the transcriptional regulation mode of anthocyanins is identical. Specifically, *RsPAP1.7c2* and *RsTT8.9c* exhibit high expression exclusively in purple tissues, thereby governing the specific upregulation of the target gene *DFR* and facilitating anthocyanin biosynthesis (Fig. 7A-B). In fleshy roots, the transcriptional regulatory gene *PAP1*, responsible for regulating anthocyanin biosynthesis, has undergone a change. Specifically, *RsPAP1.7c1* exhibits high expression exclusively in purple fleshy roots. In conjunction with *RsTT8.9c*, it governs the specific high expression of the target gene DFR, thereby driving anthocyanin biosynthesis (Fig. 7C). Interestingly, in petals, the transcriptional regulatory gene *PAP1*, responsible for regulating anthocyanin biosynthesis, has undergone another alteration. *RsPAP1.5c* is specifically highly expressed in purple petals and, in conjunction with *RsTT8.9c*, regulates the specific high expression of the target gene *DFR*, thereby driving anthocyanin biosynthesis (Fig. 7D). The findings of this study suggest that the elevated expression of the late structural genes *DFR* and *UGT* in the anthocyanin biosynthesis pathway serves as the foundation for the biosynthesis and accumulation of anthocyanins in purple tissues. Additionally, the distinct expression patterns of various copies of the *PAP1* homologous gene in the four tissues may play a crucial role in the observed color variation among different radish tissues. Further investigation and validation are necessary to fully comprehend and confirm this phenomenon.

To enhance understanding of the transcriptional regulation of anthocyanin biosynthesis in various tissues of radish, including hypocotyls, leaves, fleshy roots, and petals, quantitative real-time polymerase chain reaction (qRT-PCR) was employed to validate the central genes identified through comparative transcriptome and WGCNA. The findings indicate that the expression profiles of the identified eight regulatory hub genes involved in anthocyanin biosynthesis closely align with the patterns observed in RNA sequencing data (Fig. 7). Notably, the expression levels of the key anthocyanin biosynthesis genes *RsDFR.9c* and *RsUGT78D2.2c* were significantly elevated in purple tissues, corroborating the results obtained from RNA-seq analysis. The expression levels of the negative regulatory transcription factor *RsCPC.1c* varied among different tissues in radish, with higher levels observed in purple hypocotyls and fleshy roots compared to green/white tissues, while the opposite trend was observed in leaves and petals. In contrast, *RsTT8.9c*, a key member of the MBW transcriptional regulatory complex, exhibited significantly higher expression levels in all four purple tissues. Notably, *PAP1*, another major member of the MBW complex, has four homologous copies in radish, and its expression pattern differs across hypocotyls, leaves, fleshy roots, and petals. The expression patterns of *RsPAP1.2c*, *RsPAP1.5c*, *RsPAP1.7c1*, and *RsPAP1.2c* in various tissues were analyzed. *RsPAP1.2c* exhibited significantly higher expression in purple hypocotyls, leaves, and petals compared to green/white tissues. Conversely, *RsPAP1.5c* showed low or negligible expression levels in hypocotyls, leaves, and roots, with white petals displaying significantly higher expression than purple petals. *RsPAP1.7c1* demonstrated significantly higher expression in purple tissues compared to green tissues. *RsPAP1.7c2* exhibited significantly higher expression in purple hypocotyls and roots compared to green/white tissues, while the opposite trend was observed in leaves. These results further prove that the four homologous copies of *PAP1* play different functions in the regulation of anthocyanin synthesis in different tissues or organs of radish, and the relevant results need to be further verified.

## Discussion

The radish exhibits a broad range of planting capabilities and diverse phenotypic characteristics, rendering it suitable for consumption as sprouts, leaf vegetables, and root vegetables. Notably, certain varieties of radish, particularly those with red and purple pigmentation, possess a high nutritional value due to their abundant anthocyanin content, thereby augmenting their desirability as a food source. The availability of comprehensive genomic data pertaining to high-quality radish cultivars has facilitated the identification and documentation of numerous biological traits by researchers.

### Regulatory mechanism of color variation in different tissues of radish

In this study, a comprehensive analysis was conducted on various types of radishes, including those classified based on the color of their roots (white skin and white flesh, red skin and white flesh, red skin and red flesh, green skin and white flesh, green skin and green flesh, green skin and red flesh), as well as other variations such as yellow skin, purple skin, and black skin types. Additionally, the study also examined the variant types of radish sprouts and leaves, including those with green and purple colors. Comparative transcriptome and WGCNA analysis were performed using transcriptome sequencing data of purple-flowered and white-flowered radish, in conjunction with previously published transcriptome data of radish buds, leaves and fleshy roots. The findings of this study indicate that the late structural genes of the anthocyanin biosynthetic pathway, namely *RsUGT78D2.2c* and *RsDFR.9c*, exhibited differential expression in the purple and green/white tissues of the four radish samples. Additionally, the identification of the regulatory hub gene through WGCNA analysis revealed that *UGT78D2.2c* and *RsDFR.9c* serve as the hub gene regulating anthocyanin biosynthesis. This aligns with the previous research conducted by Muleke et al. [17], which demonstrated that *RsUFGT* plays a crucial role in regulating anthocyanin synthesis during various developmental stages of radish. *DFR* has been identified as the pivotal regulator of anthocyanin synthesis in purple leaves [23]. Extensive research has been conducted by scientific and technological experts on radish skin and radish meat in fleshy roots. The *CHS* gene has been recognized as the principal gene responsible for regulating red skin [35], whereas *RsDFR* and *RsF3H* play a crucial role in governing flavonoid synthesis in purple-skinned radish [37, 39]. In the taproot flesh, *RsDFR* and *RsLDOX* are considered indispensable for anthocyanin biosynthesis [37, 39]. In purple-skinned and purple-fleshed radish, the genes *RsCHS* and *RsDFR* have been identified as crucial regulators of anthocyanin synthesis [11]. Additionally, the high expression of *RsANS* and *RsUF3GT* has been implicated as pivotal in anthocyanin biosynthesis [8]. Overall, our findings indicate that the catalytic enzymes *CHS*, *F3H*, *DFR*, *ANS* (*LDOX*), and *UGT*, which are involved in the anthocyanin biosynthetic pathway, have been detected in various color variations across multiple tissues in radish. These genes may play a significant role in the diverse coloration observed in radish.

### Different copies of *RsPAP1* specifically regulate anthocyanin synthesis in different tissues of radish

In this study, a whole-genome identification approach was employed to identify four homologous genes (*RsPAP1.2c*, *RsPAP1.5c*, *RsPAP1.7c1*, and *RsPAP1.7c2*) belonging to the *PAP1*(*MYB75*)/*PAP2*(*MYB90*)/*MYB113*/*MYB114* family from the radish genome (Xinlimei). Subsequently, an analysis of the expression patterns revealed an intriguing phenomenon. Specifically, the expression patterns of the four copies of *RsPAP1* exhibited variations across the four different tissues of radish, with no single copy showing significant and specific up-regulation across all four tissues. Previous studies have reported the involvement of *MYB114* (*PAP1* homologous gene) in the development of purple leaves in radish, while *MYB75* (*PAP1*) is responsible for regulating anthocyanins in the red skin of radish [23, 35]. Notably, our research has revealed, for the first time, that *RsPAP1.7c2* exhibits specific and high expression levels in radish sprouts and leaves, *RsPAP1.7c1* is specifically highly expressed in purple fleshy roots, and *RsPAP1.5c* is specifically expressed in purple radish petals. In *Brassica napus*, a relative of radish, a similar phenomenon has been observed. Specifically, *BnaPAP2.A7* has been identified as a crucial regulator of anthocyanin biosynthesis in leaves [4]. Furthermore, the genes *BnaA07.PAP2*^*In−184–317*^ [34] and *BnaPAP2.A7a* [3] play a pivotal role in determining the flower color of orange-red rapeseed. Additionally, the specific upregulation of *BnaPAP2.C6a* in the purple stems of purple leaf rapeseed is essential for the synthesis and accumulation of anthocyanins in the stems [3]. In conclusion, our research findings offer novel perspectives for the subsequent investigation of the anthocyanin transcriptional regulation mechanism across various radish tissues. The tissue-specific expression of *RsPAP1*, which consequently influences the expression of *DFR* genes, could potentially account for the extensive diversity in radish coloration. Therefore, it is imperative to conduct further exploration into the regulatory mechanism underlying this phenomenon.

## Conclusion

In this study, a total of 103 genes associated with the anthocyanin biosynthetic pathway were identified from the entire radish genome. Through comparative transcriptome analysis of radish hypocotyls, leaves, fleshy roots, and petals, along with the utilization of weighted gene co-expression network analysis (WGCNA), it was determined that *RsUGT78D2.2c* and *RsDFR.9c* potentially serve as central genes involved in anthocyanin synthesis across different radish tissues. Furthermore, our analysis revealed that the tissue-specific expression of *RsPAP1*, a transcription factor responsible for regulating the expression of *DFR* genes, likely contributes significantly to the observed variations in radish coloration. These findings contribute novel insights into the understanding of anthocyanin synthesis in radish, thereby expanding our knowledge in this field.

### Supplementary Information

Supplementary Material 1.

Supplementary Material 2.

Supplementary Material 3.

Supplementary Material 4.

Supplementary Material 5.

Supplementary Material 6.

Supplementary Material 7.

Supplementary Material 8.

Supplementary Material 9.

## Data Availability

All RNA-seq data in this study were downloaded from the NCBI (https://www.ncbi.nlm.nih.gov/), with biological projects PRJNA388018 (hypocotyl), PRJNA810281 (fleshy roots), PRJNA810914 (fleshy roots). The RNA-seq data of leaves were collected from National Genomics Data Center (https://ngdc.cncb.ac.cn/gsa/browse/CRA008485) under accession ID CRA008485.
