# Supplementary figures and images for: Comparative transcriptome analysis reveals transcriptional regulation of anthocyanin biosynthesis in purple radish (Raphanus sativus L.)
Source: BMC Genomics. 2024 Jun 20;25:624. doi: 10.1186/s12864-024-10519-4 (PMC11188213; doi:10.1186/s12864-024-10519-4)

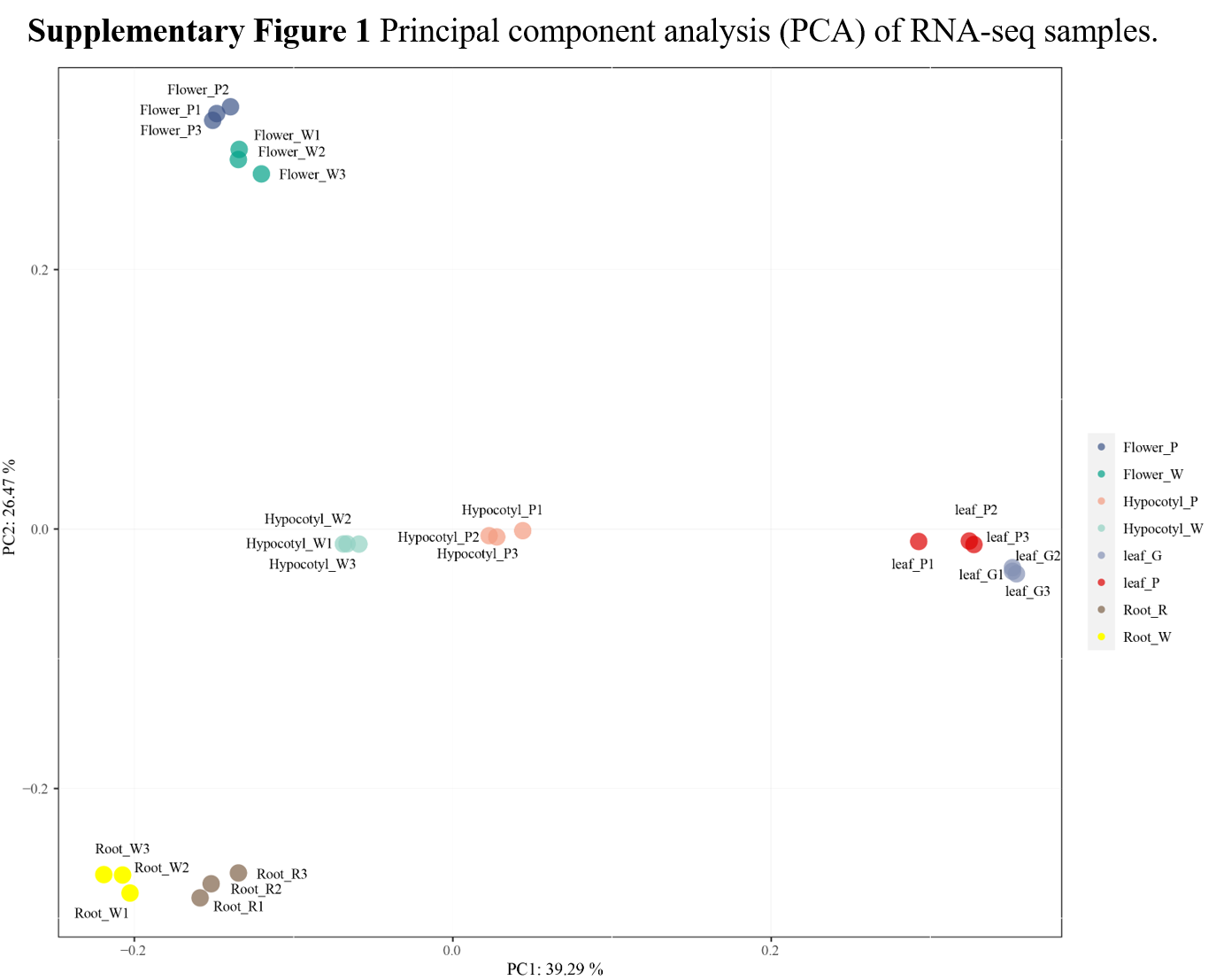

Supplement: Supplementary file 1 — Supplementary Material 1. [file 12864_2024_10519_MOESM1_ESM.tif]

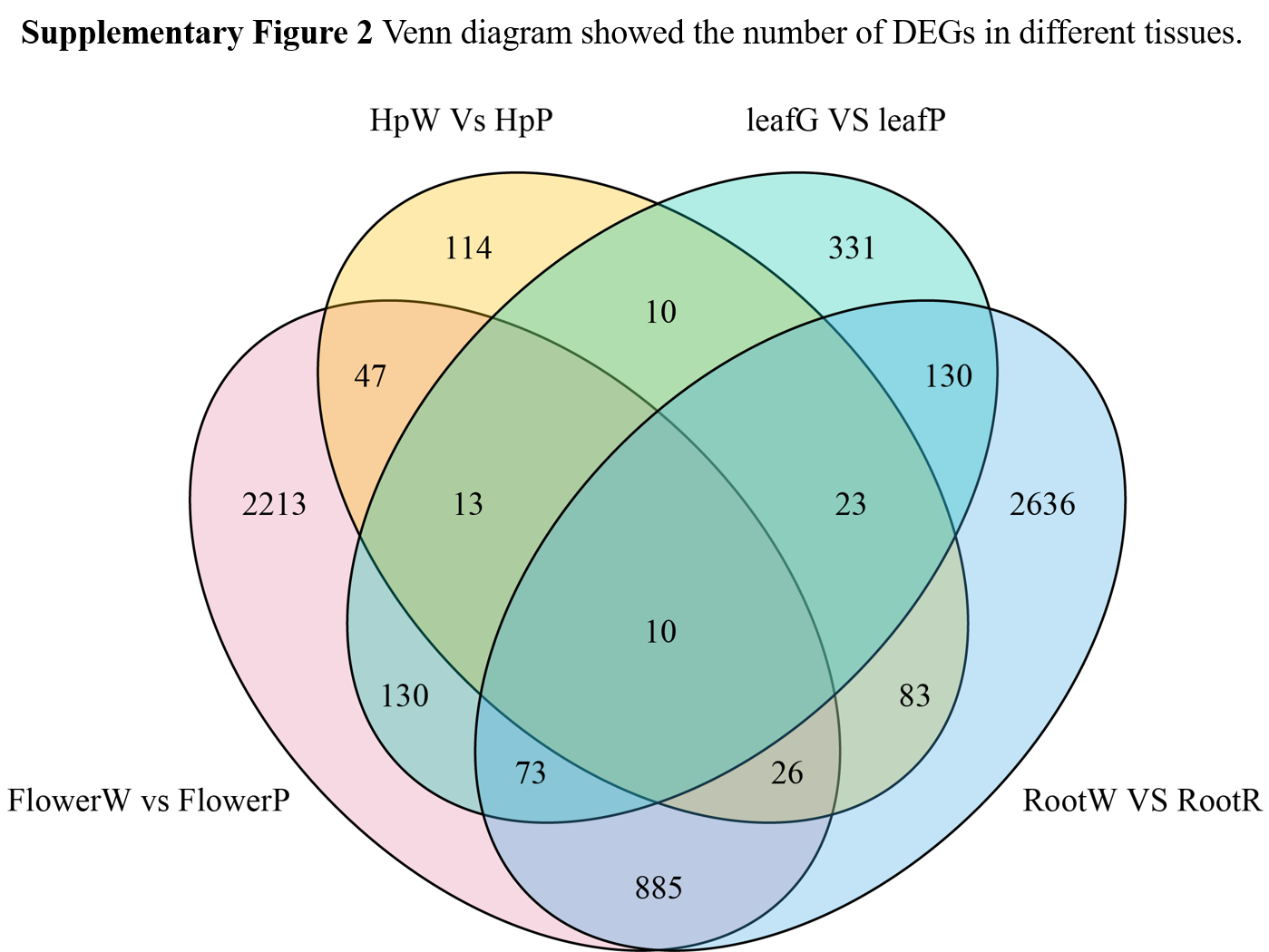

Supplement: Supplementary file 2 — Supplementary Material 2. [file 12864_2024_10519_MOESM2_ESM.tif]

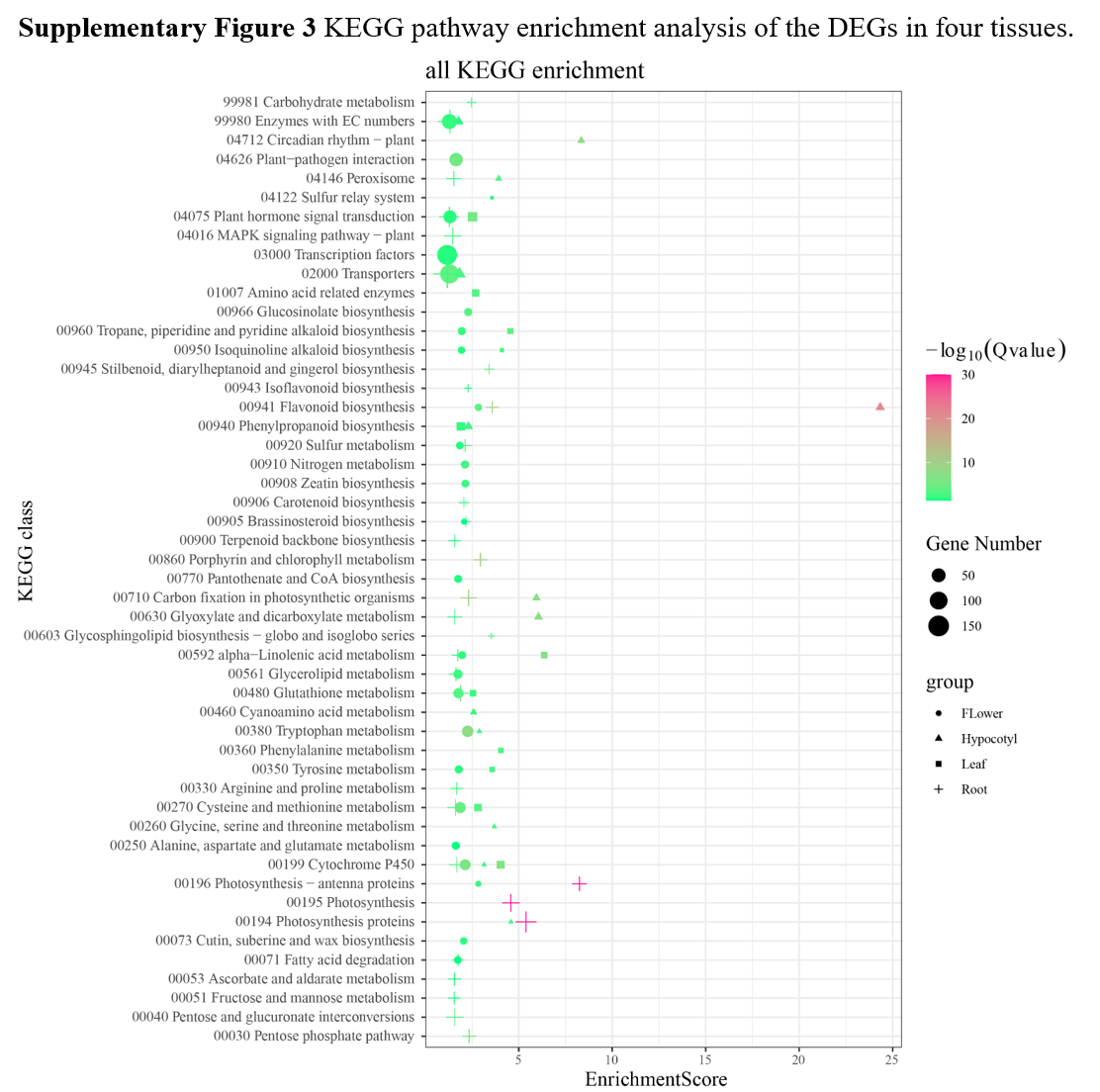

Supplement: Supplementary file 3 — Supplementary Material 3. [file 12864_2024_10519_MOESM3_ESM.tif]
